# Policy choices and compliance behavior in pandemic times

**DOI:** 10.1007/s11403-023-00380-1

**Published:** 2023-03-25

**Authors:** Giorgio Calcagnini, Slađana Pavlinović Mršić, Laura Policardo, Edgar J. Sanchez Carrera

**Affiliations:** 1 Department of Economics, Society and Politics, University of Urbino Carlo Bo, Urbino, Italy; 2 Faculty of Economics, Business and Tourism, University of Split, Split, Croatia; 3 The Customs and Monopolies Agency, Agenzia delle Dogane e dei Monopoli, Florence, Italy; 4 CIMA UAdeC, Saltillo, Mexico

**Keywords:** Evolutionary games and replicator dynamics, Infection levels, Stringency effects, Socioeconomic costs, Psychological benefits, C72, C73, H11, H12, I12, I18

## Abstract

In this paper, we model an evolutionary noncooperative game between politicians and citizens that, given the level of infection, describes the observed variety of mitigation policies and citizens’ compliance during the COVID-19 pandemic period. Our results show that different stable equilibria exist and that different ways/paths exist to reach these equilibria may be present, depending on the choice of parameters. When the parameters are chosen opportunistically, in the short run, our model generates transitions between hard and soft policy measures to deal with the pandemic. In the long-run, convergence is achieved toward one of the possible stable steady states (obey or not obey lockdown rules) as functions of politicians’ and citizens’ incentives.

## Introduction

**Fig. 1:**
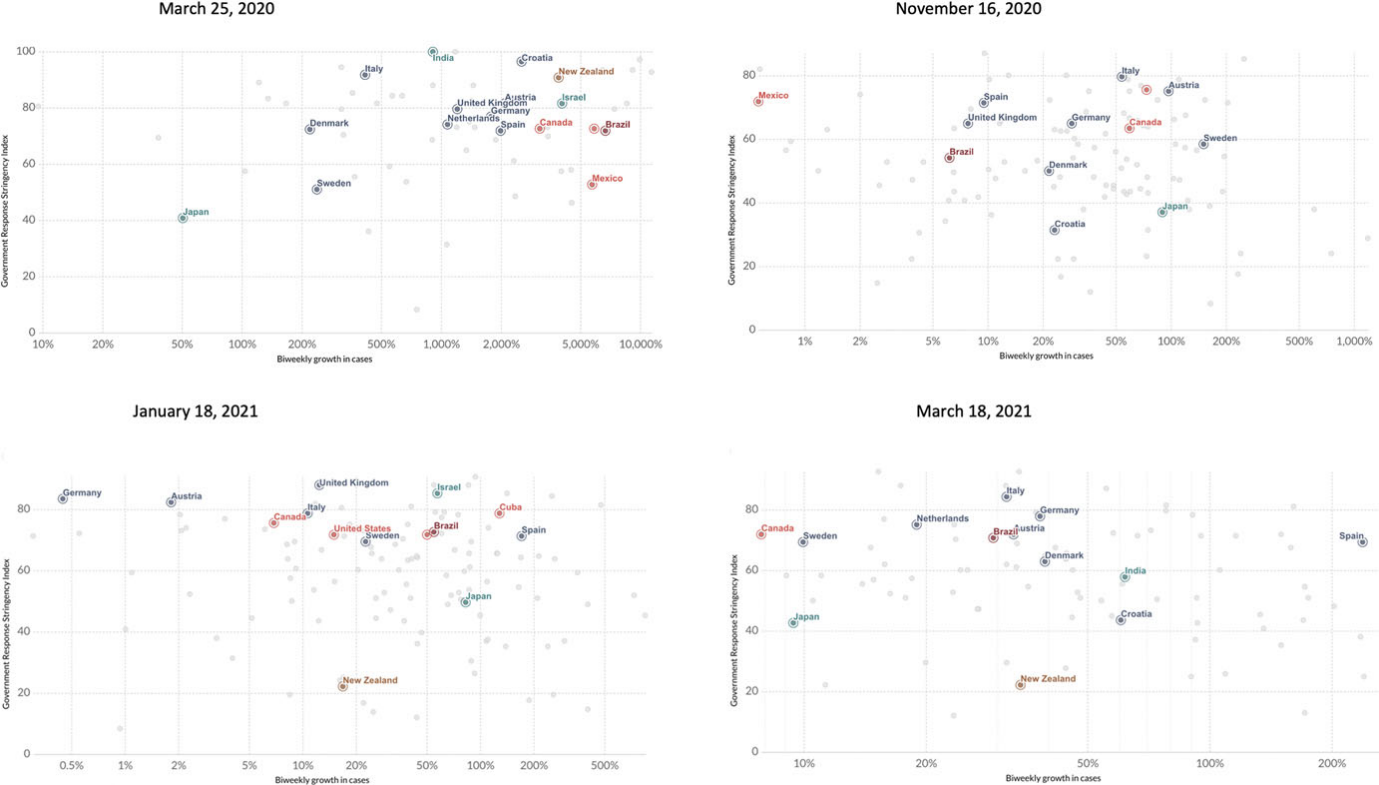
Government Response Stringency Index versus Biweekly change in confirmed COVID-19 cases. Source: Hale, Angrist, Goldszmidt, Kira, Petherick, Phillips, Webster, Cameron-Blake, Hallas, Majumdar, and Tatlow (2021). “A global panel database of pandemic policies (Oxford COVID-19 Government Response Tracker).” Nature Human Behavior. Johns Hopkins University CSSE COVID-19 Data

The outbreak of the COVID-19 pandemic prompted various, and often contradicting, mitigation responses in countries around the world (see Fig. 1). Countries with similar levels of infection rates, similar demographics and geographic characteristics often implemented very different policies to manage the pandemic [see, for example, Helsingen et al. (2020) for a comparison of the response of Sweden and Norway to the pandemic, and also Gordon et al. (2021)]. Figure 1 plots the Index of Government Stringency (GSI) for a panel of countries observed at four different dates between March 2020 and March 2021. The four quadrants of Fig. 1 show that, given an infection rate, countries adopted different strategies to deal with the diffusion of COVID-19; these mainly concerned national stringency on economic activities and citizen mobility.^1^ Moreover, given different infection rates, countries implemented similar containment measures.

This evidence, and the escalation of controversies raised by political leaders about the most suitable policies to face the COVID-19 emergency, generated in the population a feeling of mistrust about the ability of political leaders to efficiently and effectively manage the pandemic (Abbas 2020; Capano 2020; Makridis and Rothwell 2020; Tisdell 2020), with the result that their compliance to the rules depended mainly on their economic and personal incentives. For example, in September 2020 at the beginning of the second wave of pandemic, Boris Johnson publicly stated that “It is very difficult to ask the British population, uniformly, to obey guidelines in the way that is necessary,” while responding to a criticism raised by a journalist.^2^

In this paper, our model aims at investigating the relationship between politicians’ and citizens’ incentives to deal with the pandemic. It also explains why similar countries with similar levels of infection rates implemented different containment policies and why countries with different infection rates implemented similar confinement policies.^3^

The literature on the COVID-19 pandemic mainly focuses on the effectiveness of different policies aimed at controlling the diffusion of the virus, but is unable to explain the variety of policies that emerged during the time of pandemic waves.^4^ Research on COVID-19 aimed at understanding the causes and predictions of the infection trend under different policy scenarios, or the evaluation—from an empirical point of view—of its effects. For example, one branch of the COVID-19 literature assumes a benevolent social planner and, by means of an optimal control problem, studies the economic impact and the pandemic dynamic following different policies based on the classic SIR (Susceptible, Infectious, or Recovered) model or its variations (Acemoglu et al. 2021; Bischi et al. 2022; Gubar et al. 2021). This approach, however, can be criticized because it assumes the full compliance of citizens, which is not always observed in reality. Glaubitz and Fu (2020) and Reluga (2010) claimed that non-compliance is an important determinant of the pandemic evolution. Chang and Velasco (2020) slightly modified the SIR model by endogenizing the decisions of citizens to comply (or not) with lockdown policies, which in turn respond to current economic policies and expectations concerning future policies. Therefore, the infection rate pattern responds to the level of compliance (which in turn responds to economic incentives and expectations). The result is that, under the total compliance hypothesis, a perfect policy could end up being extremely ineffective, if individual economic incentives are not met.

Another branch of the literature uses a game theory approach and mitigates the hypothesis of citizens’ perfect compliance, thus investigating individuals’ incentives to comply (or not comply) with the Government’s mitigation rules. For example, McNutt (2020) applies game theory to analyze the two strategies, i.e., obey and disobey with lockdown rules, that citizens may pursue, and investigates the issue of civic disobedience during the pandemic crisis. However, in McNutt’s model, the game occurs only between citizens, while the strategies of politicians are considered/set exogenous. Similarly, in Ye et al. (2021) and Wei and Yang (2020), the dynamics of the pandemic are modeled by an underlying game that determines the behavior of citizens in the face of exogenous strategies undertaken by governments. The third branch of the literature aims at assessing the effectiveness of the variety of mitigation policies on the infection rate from an empirical point of view (Lin and Meissner 2020), or at determining which policy achieves better results (Bargain and Aminjonov 2020; Castex et al. 2020).

Our research paper is framed within the game theory literature, but diversely from previous studies, we endogenize government policy as a response to citizens’ compliance and vice versa. Politicians are assumed to be utility maximizers and their utility positively depends on the consensus they have among voters (i.e., supportive citizens) and negatively on the costs of managing the pandemic. Citizens’ utility instead depends on several factors. If they decide to obey the politicians’ rules, their utility depends on the difference between the individual’s economic evaluation of the protection they receive against the virus offered by the lockdown and the economic costs of confinement (job opportunities lost). If they decide to shirk, their utility positively depends on a “psychological effect of shirking” (which in turns positively depends on the length of the lockdown and the fraction of other disobeying citizens), and negatively on the probability of getting sick and being fined for breaking the lockdown rules.

First, we build a one-shot evolutionary game where governments decide either in favor of a soft, or a hard confinement policy as a function of obeying citizens, while citizens may choose to comply or not with the rules according to their personal and economic incentives. We then extend the model to a dynamic game between politicians and citizens and study the coevolution of their strategies over time. In the short run, we provide a rationale for why different policies implemented by countries coexist for a given level of infection rate, and why similar policies are often implemented for different infection rates.

The rest of the paper is organized as follows. Section 2 describes the setup of our one-shot population game, player payoffs, and equilibrium solutions. Section 3 develops the dynamic (evolutionary) game and studies the existence of fixed points and their stability properties. Section 3.1 discusses the results, and Sect. 4 concludes.

## Setup of the game

Let us consider a country composed by a population of politicians (*P*) and a population of citizens (*C*). Politicians, who determine the actions of the Government, observe the infection rate^5^ denoted by $I\ge 0$ and decide whether to implement a hard (*h*) or soft (*s*) policy to limit the diffusion of the infection.^6^ The lockdown policy is implemented for a given duration ${\bar{L}}\ge 1$ (the duration may be measured in days, weeks or months), and it is fulfilled (i.e., given) by both populations of politicians and citizens. Each citizen, belonging to a population *C*, observes the strategy played by politicians and decides whether to fulfill such a policy, that is to obey (*O*), or not (*NO*).

For mathematical tractability, we normalize the dimension of both populations of politicians and citizens to one. Let us denote by $x_h \in [0,1]$ the fraction of politicians choosing a hard policy and by $y_O \in [0,1]$ the fraction of obeying citizens. It follows that the fraction of politicians choosing a soft policy is equal to $(1-x_h)$ and the fraction of disobeying citizens is $(1-y_O)$.

A politician’s strategy is to decide whether to undertake a soft (*s*) or a hard (*h*) policy. Each strategy has its own associated payoff, which we denote by $\pi _i$, with $i=s,h$. The payoffs $\pi _i$, $i=s,h$ are assumed to depend negatively on the infection level and positively on the fraction of obeying citizens and on the amount of fines collected from the disobeying ones. Hence, the government’s payoffs are defined by:

1 $$\begin{aligned} \pi _h= & {} \bar{L} \left( \omega _h y_O-\alpha I \right) + F(\sigma _h (1-y_O)) , \end{aligned}$$

2 $$\begin{aligned} \pi _s= & {} \bar{L} \left( \omega _s y_O-\alpha I\right) + F(\sigma _s (1-y_O)) . \end{aligned}$$

In this model, $\alpha \in (0,1)$ is the marginal disutility given by the infection rate, irrespective of the policy chosen. Instead, the term ${\bar{L}} \omega _i y_O$, $i=s,h$ indicates the marginal increase in the politician’s payoff as the fraction of obeying citizens increases in the two different regimes (*h*) and (*s*). It can be interpreted as the marginal utility of politicians as their popularity increases among voters. Politicians’ payoff is also positively affected by the fines collected from non-obeying citizens; i.e., $F\sigma _i (1-y_O)$, where $F>0$ is the monetary value of the fine, and $\sigma _i \in (0,1)$ is the probability of being fined when the citizen decides not to obey either the policy (*h*) or (*s*). Notice that while politicians only gain popularity from obeying citizens, they can only collect fines from the non-obeying ones.

We assume that $\omega _h < \omega _s$ with $(\omega _s, \omega _h)>0$ and $\sigma _h > \sigma _s$, with $\sigma _i \in (0,1)$; $i=s,h$. The first assumption, $\omega _h < \omega _s$, reflects the fact that politicians earn higher popularity when they implement softer policies. This assumption is potentially debatable, but we believe it could be realistic in most situations. Despite cases in which this hypothesis could be criticized, such as the 2020 soft policies undertaken by Boris Johnson, that caused him a dramatic loss in popularity, in the majority of countries hard policies have been harshly criticized by the public opinion (Vlandas and Klymak 2021).^7^ Instead, the second assumption ($\sigma _h > \sigma _s$) implies that the fraction of disobeying citizens fined, is higher under a hard policy, because it is easier to discover those who do not respect the rules when all economic activities are closed down.

Let us define the payoff functions of the citizens. Citizens observe the policy $i=\{h,s\}$ implemented by the Government and its duration, and decide whether to obey or not. Citizens who decide to comply with the policy (i.e., obey) obtain a utility $\pi _O | i \in {\mathbb {R}}$, which is positively affected by *B*, i.e., citizens’ evaluation of the health benefits that the lockdown offers in terms of the reduced probability of contagion. When complying with the policy, citizens also bear a cost in terms of job opportunities lost $C_i$, which is proportional to the duration of the lockdown. We assume that *B* and $C_i$ are equal for all individuals, and that people act as rational and selfish individuals.^8^ Both the strictness of the policy and its duration are known to the citizens. Thus, the payoff functions of obeying citizens, under hard and soft lockdown regimes, are, respectively:

3 $$\begin{aligned} \pi _O|h= & {} B - C_h{\bar{L}} \end{aligned}$$

4 $$\begin{aligned} \pi _O|s= & {} \mu B - C_s{\bar{L}}. \end{aligned}$$

where $\mu \in (0,1)$ and $C_h\ge C_s$ reflect the fact that both protection against the virus and the economic costs faced by individuals are higher under hard lockdown. Non-obeying citizens, on the other hand, receive a payoff function $\pi _{NO} \in {\mathbb {R}}$, defined by:

5 $$\begin{aligned} \pi _{NO} | i= P_i({\bar{L}}, y_{O})- \theta D - \sigma _i F, \end{aligned}$$

where $P_i(\bar{L}, y_{O})$ is a psychological benefit (i.e., the “psychological effect of shirking”) associated with the type of policy $i=\{h,s\}$, which depends positively on the duration of the policy, and negatively on the number of obeying citizens. The latter are aimed at catching the “tiredness effect” and the “peer effect” that are well-known in the medical literature concerning the consequences of lockdown on individual mental health.^9^ Moreover, non-obeying citizens incur the risk of becoming infected with probability $\theta \in (0,1]$ and bearing the consequent cost (in terms of health lost) equal to $D\ge 0$.^10^ Non-compliance with the rules exposes the non-obeying individual to the possibility of being fined. They will incur fines or infringements $F>0$ as a consequence of their non-compliance with policy rules, with probability $\sigma _i \in [0,1]$. When the policy is soft and economic activities are running (even with some limitations), it is more difficult to discern whether individuals are non-obeying or if they are simply doing what they are allowed to under the softer policy. Therefore, in what follows, we will assume that for a public official it is more difficult to catch a non-obeying citizen when the policy is soft, with the consequence that $\sigma _h>\sigma _s$, and $(\sigma _h, \sigma _s)\in (0,1)$.

Note that we are assuming that citizens who do not obey with the policy do not face any economic cost. Therefore, for these citizens, $C_i = 0$, which implies that they do not receive any protection from the virus *B*. We define the psychological benefit under hard and soft lockdown as follows, i.e., $P_h(\bar{L}, y_O) = \bar{L}+\gamma (1- y_O)$ and $P_s(\bar{L}, y_O) = \beta \bar{L}+\gamma (1- y_O)$, where $\beta \in (0,1)$ means that the tiredness effect is lower when the lockdown is soft, because it decreases in proportion to the disobeying citizen’s payoff during soft lockdown. Moreover, the tiredness effect is greater in proportion to the duration of the lockdown (i.e., the greater the effect of the psychological shirking benefit of the non-obeying citizen, the longer the duration of the policy $\bar{L} \ge 1$). Parameter $\gamma \ge 0$ measures peer effects. Indeed, disobedience toward the policy—while all other citizens are obeying—does not produce any increase in the psychological benefit of shirking. That is to say, the psychological benefit from non-obeying the policies increases as more and more citizens do not obey such policies. Therefore, the payoff of non-obeying citizens under a hard and a soft policy is:

6 $$\begin{aligned} \pi _{NO}|h= & {} \bar{L}+\gamma (1- y_O)- \theta D - \sigma _h F \end{aligned}$$

7 $$\begin{aligned} \pi _{NO}|s= & {} \beta \bar{L}+\gamma (1- y_O)- \theta D - \sigma _s F \end{aligned}$$

The equation for the psychological shirking benefit is supported by the literature. In the behavioral literature, Tunçgenç et al. (2021:13) find that “the best predictor of people’s adherence to distancing was perceived adherence of their close circle, which exceeded the effect of people’s own approval of the rules,” so as to corroborate our hypothesis. Tuncgenc et al. (2021) conducted a global study through a survey held from April to June 2020, involving 6500 participants. They asked people how much they, their friends, and their fellow citizens approved of and observed the COVID-19 guidelines in their country. This is crucial, as people are more likely to accept new social rules when they have high expectations that others are following those rules as well. Their findings do not support the individualist assumptions of many governments: People who followed guidelines the most were not those who found the rules more justified, or those who were more vulnerable to the disease. The most diligent rule followers were, consistently, those whose friends and families were following the rules. Further, people who were more vulnerable to COVID-19 were more likely to follow the guidelines if they had a wide social circle. These findings applied across age groups, genders, countries, and were independent of the strength of local restrictions. Indeed, in the medical literature some authors found a positive association between perceived changes in everyday life and a more significant decrease in social contacts, with higher mental health impairments (Christoph et al. 2020). Moreover, Murphy et al. (2020) found that “people become less compliant, the longer they had experienced lockdown.” Additionally, a common sentiment that circulates among entrepreneurs, after long lockdown periods, was that in spite of all the restrictions imposed by the government, it might be better to ignore them and risking getting fined, than closing down all activities.

### Threshold levels and optimal strategies

Let us compute the expected payoffs. With some easy calculations, the expected payoff of a politician under hard lockdown is given by Eq. 8, and the soft lockdown expected payoff is given by Eq. 9. That is, for politicians, the expected payoffs from hard and soft policies are:

8 $$\begin{aligned} E_{P_h}= & {} y_O\pi _h+(1-y_O)\pi _h= \bar{L} \left( \omega _h y_O-\alpha I\right) + F(\sigma _h (1-y_O)) \end{aligned}$$

9 $$\begin{aligned} E_{P_s}= & {} y_O\pi _s+(1-y_O)\pi _s= \bar{L} \left( \omega _s y_O-\alpha I\right) + F(\sigma _s (1-y_O)). \end{aligned}$$

which are weighted averages of the payoffs under the two policies, and the weights are the number of obeying and disobeying individuals. Comparing the expected payoffs of hard and soft policies, politicians will choose a hard lockdown if $E_{P_h} > E_{P_s}$; otherwise, they will choose a soft one. Therefore, their choice depends on the fraction of obeying citizens $y_O$, because politicians will choose this strategy *h* if and only if the fraction of obeying citizens is lower than a given threshold value, i.e., $y_O < y^*_O$, where

10 $$\begin{aligned} y^*_O= \frac{F(\sigma _h - \sigma _s)}{L(\omega _s-\omega _h) + F(\sigma _h - \sigma _s)} . \end{aligned}$$

Recall that $\sigma _h>\sigma _s$ and $\omega _s>\omega _h$, so $0\le y^*_O \le 1$. Hence, politicians’ decision to choose a hard or a soft policy is based on the ratio between the additional fines collected under a hard policy regime (the numerator of equation 10) and the trade-off between the excess fines collected and the popularity lost by politicians (the denominator of equation 10). Equations 1 and 2 show that politicians only obtain additional popularity from obeying citizens with a soft policy ${\bar{L}}(\omega _s-\omega _h)$. Further, additional fines under a hard policy, compared to a soft policy, $F(\sigma _h-\sigma _s)$, are only collected from non-obeying citizens. If the fraction of obeying citizens is above the threshold $y^*_O$, additional popularity of a soft policy prevails on politicians’ utility over additional fines collected under a hard policy. The opposite holds if the fraction of obeying citizens is below $y^*_O$. Therefore, when the fraction of obeying citizens is lower than $y_O^*$, politicians will implement a hard policy; otherwise, they will implement a soft policy.

For citizens, the expected payoffs of the two strategies, i.e., obey and non-obey, are:

11 $$\begin{aligned} E_{C_O}= & {} x_h\left( B- C_h{\bar{L}}\right) +(1-x_h)\left( \mu B -C_s{\bar{L}} \right) \end{aligned}$$

12 $$\begin{aligned} E_{C_{NO}}= & {} x_h\left( {\bar{L}}+\gamma (1- y_O) -\theta D -\sigma _h F\right) + (1-x_h)\left( \beta {\bar{L}}+\gamma (1- y_O) -\theta D -\sigma _s F \right) \nonumber \\ \end{aligned}$$

Citizens will choose the obey strategy (*O*), instead of non-obey strategy (*NO*), if and only if $E_ {C_O} \ge E_ {C_ {NO}}$. This is the case when the fraction of politicians choosing *hard* $x_h$ is below or beyond a given threshold $x^*_h$, where

13 $$\begin{aligned} x^*_h= \frac{-{\bar{L}}(\beta + C_s) +\theta D +\mu B - (1-y_O)\gamma +\sigma _s F}{{\bar{L}}(1-\beta ) + B(\mu -1)+{\bar{L}}(C_h-C_s)-F(\sigma _h-\sigma _s)}. \end{aligned}$$

In what follows, we will consider and make use of the following definitions:

14 $$\begin{aligned} K= & {} {\bar{L}}(\beta + C_s) - \mu B - \theta D - F \sigma _s \end{aligned}$$

15 $$\begin{aligned} W= & {} {\bar{L}}(1 + C_h) - B - \theta D - F \sigma _h \end{aligned}$$

16 $$\begin{aligned} DF= & {} F(\sigma _h - \sigma _s) \end{aligned}$$

17 $$\begin{aligned} DL= & {} {\bar{L}} (\omega _s - \omega _h) \end{aligned}$$

meaning that *K* is the excess utility of non-obeying over obeying under a soft policy, when the peer effect is assumed to be zero. It can be rewritten as $K=\pi _{NO}|s - \pi _O|s - \gamma (1-y_O)$. The term *W*, on the other hand, represents the same measure under strict policy and can be rewritten as $W=\pi _{NO}|h - \pi _O|h - \gamma (1-y_O)$. The term *DF* is the excess of fines collected under a hard policy as opposed to a soft lockdown, and *DL* is the excess popularity enjoyed by adopting a soft lockdown policy versus a hard policy. (From another perspective, it can be read as the popularity lost by applying a hard policy relative to the popularity that could have been achieved by applying a soft lockdown policy.)

The threshold level of obeying citizens $y_O^*$ represented by Eq. 10, in which politicians find it optimal to adopt a hard policy, and by using Eqs. 16–17, can be rewritten as:

18 $$\begin{aligned} y^*_O= \frac{DF}{DF+DL} . \end{aligned}$$

Similarly, by means of Eqs. 14–15, the threshold 13 may be rewritten as:

19 $$\begin{aligned} x^*_h= \frac{-K - \gamma (1-y_O)}{W-K} \end{aligned}$$

with $0\le x^*_h \le 1$. The term $x^*_h$ can be interpreted as the fraction between the excess payoff of obeying with respect to non-obeying under a soft policy (the numerator) and the trade-off between non-obeying and obeying under a hard and a soft policy (the denominator). Therefore, while the numerator is affected by the peer effect, the denominator is not. Given that *W* and *K* can be either positive or negative, and seeing that $x^*_h$ must necessarily be positive and fall between 0 and 1, two cases follow. However, depending on the sign of the denominator in equation 19, different scenarios may arise.

1. ***Case 1***. Both the numerator and denominator of 19 are negative, which implies that citizens choose to obey if the fraction of politicians choosing a hard policy is greater than the threshold, $x_h >x_h^*$, i.e., the excess utility of disobeying with respect to obeying under a hard lockdown is lower than in a soft one. This case divides into four subcases:
  **Case 1a**: $W<0$ and $K>0$. The trade-off between *obey* and *disobey* under a *hard* policy is positive, while it is negative under a *soft* policy. When politicians choose *hard*, citizens’ dominant strategy is *obey*, while if politicians choose *soft* citizens’ dominant strategy is *disobey*. This is a case in which the reduced probability of being fined under a *soft* policy makes it easier for people to break the rules in order to avoid the policy cost.
  **Case 1b**: $W<0$, $|W|>|K|$, $K<0$ and $-K<\gamma (1-y_O)$. The trade-off between *obey* and *disobey* is positive both in the presence of hard and soft policies, but the excess utility of *obey* with respect to *disobey* in *soft* is smaller than the peer effect. Therefore, in the case of a *soft* policy, if the number of obeying individuals is “large enough,” the peer effect may prevail over *K* and *disobey* is the citizens’ dominant strategy.
  **Case 1c**: $W>0$, $K>0$ $|K|>|W|$. The citizens’ dominant strategy is *disobey* both under hard and soft policy regimes. This result holds irrespectively of the peer effect value.
  **Case 1d**: $-W=\gamma$ and $K=0$. The trade-off between *disobey* and *obey* is zero at point (0,1), i.e., *soft* and *obey*. At point (1,0), i.e., *hard* lockdown and *disobey*, the excess utility of *disobey* with respect to *obey* (net of the peer effect) is exactly equal to the peer effect. In other words, this is case in which citizens are indifferent toward the two strategies.
2. ***Case 2***. Both the numerator and denominator of 19 are positive, which implies that citizens choose to *obey* if the share of politicians choosing *hard* is lower than the threshold, $x_h \le X_h^*$. Here, the excess utility of *obey* with respect to *disobey* under a soft policy is positive and greater than the peer effect. Therefore, the citizens’ dominant strategy in the case of a soft policy is to *obey*, irrespectively of the number of disobeying citizens. Instead *W* can be positive or negative. ***Case 2*** can be divided into two subcases:
  **Case 2a**: $K<0$, $|K|>\gamma (1-y_O)$ and $W>0$. *Disobey* is the dominant strategy in the presence of a hard policy, while *obey* is the dominant strategy under a soft policy; this is true since *K* prevails, in absolute value, over the peer effect.
  **Case 2b**: $K<0$, $|K|>\gamma (1-y_O)$, $W<0$ and $|W|<|K|$. *Obey* is the dominant strategy for citizens under soft and hard policies, if the peer effect is zero. However, if $\gamma >0$, the peer effect could prevail over *W*, since $|W|<|K|$. So, if the number of disobeying citizens is “large enough,” *disobey* is the dominant strategy for individuals under a hard policy.

From Eq. 19, $x^*_h$ is a function of $y_O$ due to the peer effect $\gamma (1-y_O)$. It follows that, depending on the fraction of obeying citizens, multiple equilibria may arise. We will extend this static game to a dynamic one in the next Section. Further, we will study the replicator dynamics, the existence of its fixed points, its stability properties and the dynamics surrounding/accompanying them.

## The evolutionary game

Let us consider politicians’ expected payoffs from the strategies *hard* and *soft* as a function of the share of obeying citizens, as in Eqs. 8 and 9. It follows that the average payoff for politicians can be calculated as a weighted average of the expected payoffs from the two strategies. That is, the average payoff of politicians is:

20 $$\begin{aligned} {\bar{E}}^P=x_h(E_{P_h}) + (1-x_h)(E_{P_s}). \end{aligned}$$

Similarly, given the citizens’ expected payoffs for strategies *obey* and *disobey*, as in Eqs. 11 and 12, we can compute the citizens’ average payoff, i.e.,

21 $$\begin{aligned} {\bar{E}}^C=y_O(E_{C_O}) + (1-y_O)(E_{C_{NO}}). \end{aligned}$$

The Replicator Dynamic (RD) in continuous time (Sandholm, 2010; Weibull, 1995) of the strategies *hard* and *obey* is therefore represented by the following system of two differential equations,^11^

22 $$\begin{aligned} \left\{ \begin{array}{l} {\dot{x}}_{h}=x_h \left[ E_{P_h}- \bar{E}^P\right] =x_h(1-x_h)\left[ E_{P_h}- E_{P_s} \right] \\ \\ {\dot{y}}_{O}=y_O \left[ E_{C_O}- \bar{E}^C\right] = y_O (1-y_O) \left[ E_{C_O}- E_{C_{NO}}\right] \end{array} \right. \end{aligned}$$

or, if we substitute Eqs. 8, 9, 11 and 12 into 22 and rearrange them as follows,

23 $$\begin{aligned} \left\{ \begin{array}{l} {\dot{x}}_{h}=x_h(1-x_h)\left[ {\bar{L}}y_O(\omega _s-\omega _h) + F(1-y_O)(\sigma _s-\sigma _h)\right] \\ \\ {\dot{y}}_{O}=y_O (1-y_O)\left[ x_h(B-C_h{\bar{L}})+ (1-x_h)(\mu B-C_s{\bar{L}})-{\bar{L}}(\beta -x_h(1-\beta ))+\theta D-\right. \\ \left. \quad \quad \gamma (1-y_O)+F(\sigma _hx_h+\sigma _s(1-x_h)) \right] ] \end{array} \right. \nonumber \\ \end{aligned}$$

The RD system indicates that the number of agents adopting a given strategy increases if the expected payoff of that strategy is greater than the average payoff attained by the population they belong to, otherwise it decreases. This RD system (23) is a nonlinear two-dimensional dynamical system in continuous time. The first step to understanding its qualitative dynamic behavior is to investigate the existence of fixed points in the action space of the players (i.e., the Cartesian $[0, 1] \times [0, 1]$ plane), as well as their local stability properties. Setting ${\dot{x}}_h =0$ and ${\dot{y}}_O=0$ and solving for $x_h$ and $y_O$, it is possible to prove the existence of at most 7 fixed points (depending on the set of parameters chosen) in the players’ action space ($[0,1] \times [0,1]$ Cartesian plane), each one with its own stability properties. “Appendix” herein contains the computation of all the seven fixed points and an analysis of their stability properties.

As shown in “Appendix,” the fixed points that exist in this system (23) are the four corner solutions, that is (0, 0), (1, 0), (0, 1) and (1, 1), i.e., two border fixed points,^12^ which we define as $E_{b1}$ and $E_{b2}$ and are equal to:

$$\begin{aligned} E_{b1}= & {} \Bigg (0, \frac{K+\gamma }{\gamma } \Bigg ), \\ E_{b2}= & {} \Bigg (1, \frac{W +\gamma }{\gamma } \Bigg ), \end{aligned}$$

and an interior solution $(x^*_h, y^*_O)$.

From the analysis of the stability properties of the seven different equilibria, it is possible to prove that, under certain configurations of the parameters, only two equilibrium points can be considered asymptotically stable, at least “in most cases.”^13^ These two stable equilibria that arise from the model are (0, 1); that is, all politicians choose a soft policy and all citizens obey, and (1, 0); namely, all politicians choose a hard policy and all citizens disobey. From a policy perspective, it is also interesting to study the global dynamics involved in the interior equilibrium, which may provide insight into the rationale of politicians who want to induce the system to converge toward one of the preferred stable equilibria (provided there are multiple stable fixed points).

The first stable equilibrium (0, 1) is the one characterized by the soft policy chosen by policymakers, with all the citizens obeying. As is discussed in the “Appendix,” a sufficient condition for stability of this equilibrium is that $K<0$, i.e., that the dominant strategy for citizens under *soft* is to obey. Citizens’ dominant strategy under a hard policy can be either *obey* or *disobey*, depending on the value of *W*, that can either be positive or negative.

The second stable equilibrium is (1, 0) when all politicians choose to adopt a hard policy and all citizens disobey. This equilibrium is characterized by the fact that $-W<\gamma$; in other words, the excess utility of *disobey* with regard to *obey* (*W*), net of the peer effect, can be positive or, alternatively, negative. However, it will be smaller than the peer effect.

The dynamics leading to those different equilibria, however, may be different depending on the parameters of the problem case (1 or 2) in which we find ourselves; subsequently, the subcase we are in is also determined.

In this section, a graph illustrates the diverse dynamics that arise in the six different scenarios; we accompany this with a discussion thereof. To simulate the dynamics of the model in the six different cases, we selected a set of feasible parameters for each case and plotted their corresponding dynamics. The parameters used to simulate the different dynamics are those listed in Table 1.

**Table 1:** Configuration of the parameters for the simulations

Parameter	Case 1a	Case 1b	Case 1c	Case 1d—Bifurc	Case 2a	Case 2b
B	10	10	10	10	10	10
D	9	9	1	9	3	3
$C_h$	7.5	7.5	7.5	7.25	5.6	5.4
$C_s$	7.3	6.6	6.6	7.2	2.75	2.75
$\mu$	0.7	0.7	0.7	0.7	0.7	0.5
L	2	2	2	2	2	2
$\beta$	0.7	0.7	0.7	0.7	0.5	0.5
$\theta$	0.9	0.9	0.9	0.9	0.5	0.5
$\gamma$	6.5	6.5	2	6.5	0.5	0.5
F	7	7	7	7	2	2
$\sigma _h$	0.7	0.7	0.2	0.7	0.7	0.7
$\sigma _s$	0.1	0.1	0.1	0.1	0.2	0.2
$\omega _s$	2	2	2	2	0.7	0.7
$\omega _h$	0.4	0.4	0.7	0.4	0.3	0.3
W	– 6	– 6	4.7	$-$ 6.5	0.3	$-$ 0.1
K	0.2	$-$ 1.2	6	0	$-$ 2.4	$-$ 0.4
W-K	$-$ 6.2	$-$ 4.8	$-$ 1.3	$-$ 6.5	2.7	0.3

**Fig. 2:**
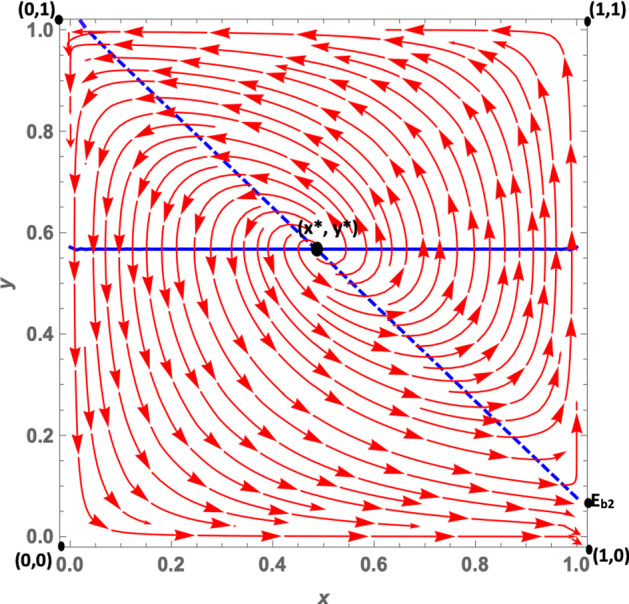
Phase plane of the RD system corresponding to case 1a. The blue dashed line is the nullcline ${\dot{y}}_{O}=0$, and the straight blue line is the nullcline ${\dot{x}}_{h}=0$ (color figure online)

**Fig. 3:**
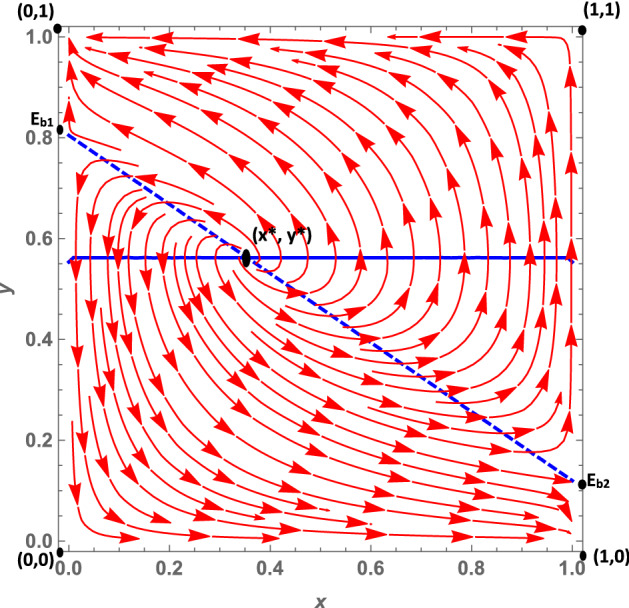
Phase plane of the RD system corresponding to case 1b. The blue dashed line is the nullcline ${\dot{y}}_{O}=0$, and the straight blue line is the nullcline ${\dot{x}}_{h}=0$ (color figure online)

**Fig. 4:**
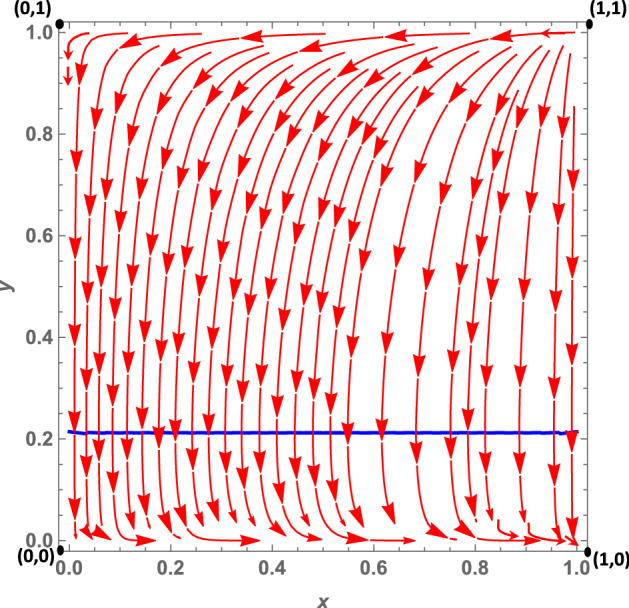
Phase plane of the RD system corresponding to case 1c. The straight blue line is the nullcline ${\dot{x}}_{h}=0$ (color figure online)

**Fig. 5:**
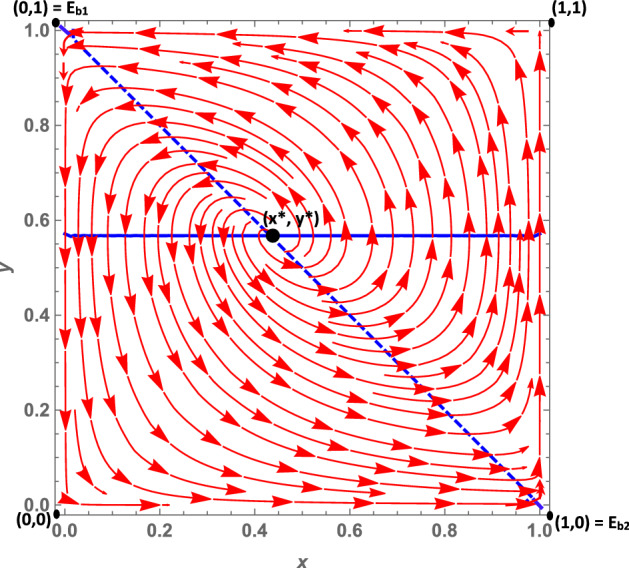
Edge-corner Bifurcation corresponding to case 1d. Phase plane of the RD system. Edge-to-corner bifurcations occur. The blue dashed line is the nullcline ${\dot{y}}_{O}=0$, and the straight blue line is the nullcline ${\dot{x}}_{h}=0$ (color figure online)

**Fig. 6:**
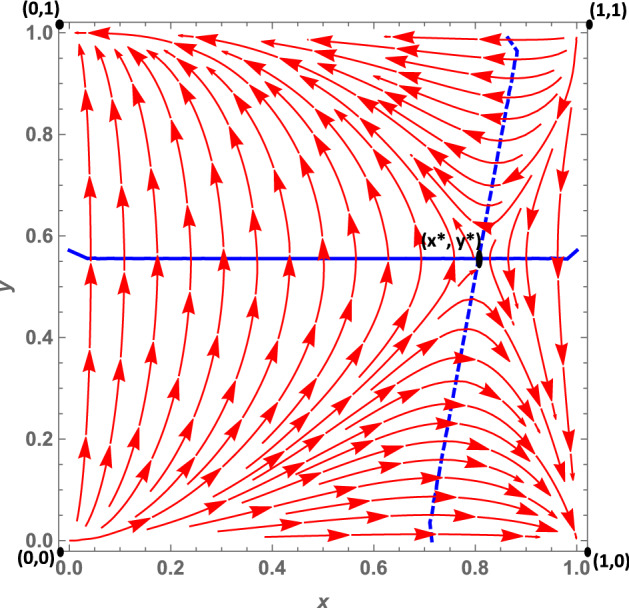
Phase plane of the RD system corresponding to case 2a. The blue dashed line is the nullcline ${\dot{y}}_{O}=0$, and the straight blue line is the nullcline ${\dot{x}}_{h}=0$ (color figure online)

**Fig. 7:**
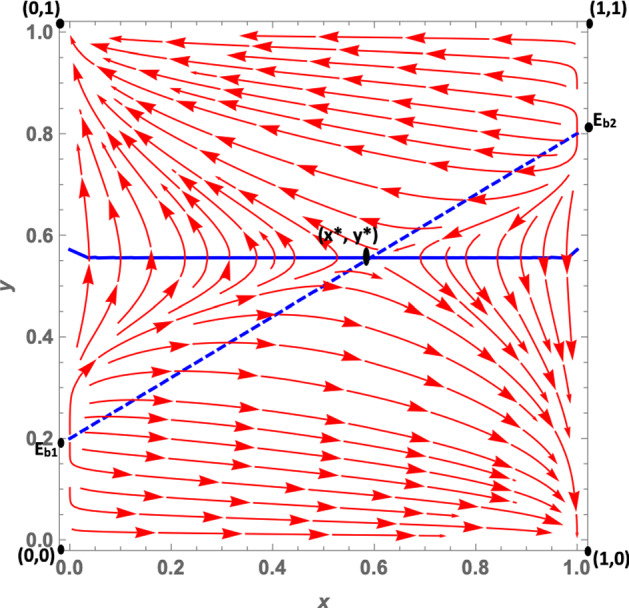
Phase plane of the RD system corresponding to case 2b. The blue dashed line is the nullcline ${\dot{y}}_{O}=0$, and the straight blue line is the nullcline ${\dot{x}}_{h}=0$ (color figure online)

Let us analyze the dynamics case by case:

- **Case 1a**: As we mentioned in Sect. 2.1, this case is characterized by the fact that citizens’ dominant strategy under a soft policy is *disobey*, as suggested by $K>0$, while the dominant strategy under a hard policy (net of the peer effect) is *obey*. The peer effect, however, is fairly large ($\gamma =6.5$) and may offset *W*, when the number of disobeying citizens is large enough.^14^ It follows that when the number of non-obeying citizens increases, the dominant strategy under a hard policy may become *disobey*. This leads to the dynamic shown in Fig. 2: The dynamics are represented by a growing spiral starting from the internal equilibrium, which converges to the only stable fixed point represented by (1, 0). Indeed, if we assume that *obey* and *hard* are frequent strategies (so that we are at a point north-east of the interior equilibrium), politicians will try to maximize their utility by choosing a soft policy, because this allows them to gain utility from increased popularity. Diversely, if politicians choose a soft policy, citizens’ dominant strategy is *disobey*, so $y_O$ decreases. At this point, since the number of obeying citizens decreases, fines become more important for politicians; thus, they will try to maximize their utility by choosing a hard policy. However, at this point the dominant strategy for citizens becomes *obey*, and as long as the share of disobeying citizens is not very large, this remains the dominant strategy. As a consequence, the system continues rotating around the internal equilibrium, until it reaches the stable state (1, 0). Indeed, given this set of parameters, the eigenvalues associated with the equilibrium (0, 1) are one positive and one negative. However, those associated with the (1, 0) equilibrium are all negative, so that the system converges toward this equilibrium point. (More details can be found in “Appendix.”)
- **Case 1b**: *W* and *K* are both negative. Therefore, *obey*, net of the peer effect, is a dominant strategy under both hard and soft lockdowns. However, the peer effect is quantitatively large and may offset the excess utility of *obey* compared with *disobey*, even when the share of disobedient citizens is low. The size of the peer effect is particularly visible when the policy implemented is a soft lockdown. When the lockdown is *hard*, the share of disobeying citizens must be quantitatively significant so that the peer effect offsets *W*. This case is shown in Fig. 3. Assuming that *obey* and *hard* policies are frequent strategies, such as at a point north-east of the interior equilibrium, politicians will try to maximize their popularity, given that the share of obeying individuals is larger than $y_O$. However, since $K<0$, citizens’ choice of *obey* becomes easier to make, and as long as the number of obeying citizens is large enough, i.e., $-K>\gamma (1-y_O)$, the dominant strategy for citizens remains *obey* and the system converges toward (0, 1). In other words, all the politicians choose a soft policy and all the citizens *obey*. Diversely, when *hard* and *obey* are rare strategies, such as at a point south-west of the interior equilibrium, politicians will try to maximize their utility by imposing fines. Therefore, they will choose a hard policy since the number of obeying individuals is small. But for citizens, the dominant strategy under *hard* is to *disobey*, since the number of disobeying citizens is large and the (absolute value of the) peer effect $\gamma (1-y_O)$ offsets *W*. This leads the system to converge toward (1,0) where all politicians choose a hard policy and all citizens *disobey*. The set of parameters *W* and *K* both negative leads to the local stability of both equilibria (1,0), that is, *hard* and *disobey*, and (0,1), that is, *soft* and *obey*. Further, the eigenvalues of the first (1,0) equilibrium are both negative ($\lambda _1 = -DF$ and $\lambda _2 = -W-\gamma$), and so are the eigenvalues of the second (0,1) equilibrium ($\lambda _1 = -DL$ and $\lambda _2 = K$) (see “Appendix”).
- **Case 1c**: *W* and *K* are both positive. Therefore, the dominant strategy under both soft and hard lockdowns is *disobey*, irrespective of the peer effect value. Furthermore, *W* is greater than *K*, i.e., the excess utility of *disobey* with respect to *obey* is greater under *hard* than *soft*. This case basically represents a situation in which it is never easier for citizens to respect policy rules; this is because the economic costs avoided and the psychological benefits of disobeying are greater than the potential benefits of not getting infected by complying with the confinement rules. Costs would include the risk of health loss due to the chance of contracting the virus, and the potential costs of fines. This scenario leads to a dynamic shown in Fig. 4. In this case, the equilibrium represented by *soft* and *obey* is locally unstable. Its associated eigenvalues are $\lambda _1= -DL$ and $\lambda _2 = K$, which are negative and positive, respectively (see “Appendix”). Therefore, equilibrium (0, 1) is a saddle point. The equilibrium represented by *hard* and *disobey*, instead, has eigenvalues equal to $\lambda _1=-DF$ and $\lambda _2=-W-\gamma$, which are both negative. Furthermore, whatever the initial conditions are, the system will converge, sooner or later, to the equilibrium (1, 0), i.e., to *hard* and *disobey*.
- **A special case 1d**: edge-to-corner bifurcation. It is worth noting that the equilibria (0, 1) and (1, 0) change their stability properties as a consequence of a bifurcation. When $E_{b_1}$ and $E_{b_2}$ are included in the player action space, then the equilibria (0, 1) and (1, 0) are stable. For specific sets of parameters when $K=0$ and $-W=\gamma$, it may occur that $(0,1)=E_{b_1}$ and $(1,0)=E_{b_2}$ (the boundary equilibrium disappears through the transcritical bifurcation, despite the fact that these conditions are not necessarily simultaneously met), and equilibrium (0, 1) inherits the stability properties of $E_{b_1}$ and equilibrium (1, 0) those of $E_{b_2}$. When both bifurcations simultaneously occur, i.e., when $(0,1)=E_{b_1}$ and $(1,0)=E_{b_2}$, none of the equilibria are locally stable. (All the equilibria have at least one nonnegative eigenvalue at their associated Jacobian matrix.) Therefore, this special case is characterized by a perpetual dynamic characterized by a diverging spiral starting from the internal equilibrium. From an economic point of view, as we suggested in Sect. 2.1, in this case the excess utility of *obey* with respect to *disobey* (net of the peer effect) is zero, and a situation in which the excess utility of *obey* (net of the peer effect) under *hard* is equal to the peer effect. Herein, citizens diverge from the equilibrium (0, 1), i.e., *soft* and *obey*, because they are indifferent toward the two strategies. Since disobeying citizens do not exist, $K=0$ means that no strategy is dominant at the (0, 1) point. This set of parameters also makes the other equilibrium (1, 0), i.e., *hard* and *disobey*, unstable (Fig. 5). This is true given that—at this point, since all citizens are disobeying the rules—no strategy is dominant. Indeed, the excess utility of *obey* with regard to *disobey* ($-W$) is exactly equal to the peer effect. (The equality $-W=\gamma$ is verified.) Thus, once again, citizens are indifferent toward the two strategies.
- **Case 2a**: In this case, as mentioned in Sect. 2.1, $W-K$ is positive and this leads to two real eigenvalues associated with the Jacobian computed at the interior equilibrium point. *W* is positive, and therefore citizens’ dominant strategy under *hard* is *disobey*, irrespective of the peer effect, while the dominant strategy under a soft policy, net of the peer effect, is *obey*. (Indeed *K* is negative.) The peer effect is small compared to the value of *K*, therefore it never offsets the excess utility of *obey* with respect to *disobey*. Further, it implies that under a soft policy the dominant strategy for citizens is *obey*. This situation gives rise to the dynamics shown in Fig. 6. Whatever the initial conditions are, the system will rapidly converge to either (0, 1) or (1, 0). The basin of attraction of the equilibrium (0, 1) is quite large, consisting in more than half of the surface of the action space representing the set of feasible actions.
- **Case 2b**: In this case, as pointed out in Sect. 2.1, citizens’ dominant strategy (net of the peer effect) under both hard and soft lockdowns is *obey*; however, the peer effect may offset both *K* and *W* if the number of disobeying citizens becomes large enough. Therefore, assuming that *hard* and *obey* are frequent strategies, politicians will try to maximize their utility by seeking greater popularity from the obeying individuals; thus they will choose *soft*. Two cases exist. First of all, if the number of obeying individuals is large enough and the peer effect does not offset the excess utility of *obey* with respect to *disobey* under *soft*, the system will converge toward (0, 1). Secondly, instead—if the number of obeying individuals is small—then the peer effect will offset *K* so that citizens will start disobeying. As long as the number of disobeying citizens increases, politicians will try to increase their payoff by imposing fines and will therefore choose a hard policy. But when the number of disobeying citizens is large, the peer effect offsets *W* in absolute value, and citizens’ dominant strategy will become *disobey*. The system will end up converging toward (1, 0), i.e., to a situation in which all the politicians choose a hard policy and all the citizens *disobey*. This dynamic is represented in Fig. 7.

### Discussion

Results from the analysis of the six scenarios may provide thought-provoking/attractive hints for policy makers. The different dynamics governing the convergence to one of the equilibria, depending on which of the previous cases we consider, can somehow be interpreted as a “short-term” response to the actions undertaken either by the politicians’ and citizens’ populations. The “long-run” response is, of course, represented by the stable fixed point to which the system (i.e., the populations) converges.

Only incentives can determine short-term responses and, therefore, the system dynamics in disequilibrium, as well as the convergence to one of the possible stable fixed points.

The short-term dynamics that arises from our model sheds light on the reasons for which, during the COVID-19 pandemic that occurred between 2020 and 2021, we observed countries facing similar levels of infection but responding very differently in terms of containment levels.

The analysis performed identified two broad scenarios that are characterized by very different dynamics. The first, indicated by case 1 in Sect. 2.1, which generates oscillations around the internal equilibrium, gives rise to a diverging spiral. The second, indicated by case 2, which generates a uniform path toward one of the two stable fixed points, depending on the initial conditions. The number of fixed points and their stability properties may vary, depending on the scenario.

The second case (which includes scenarios 2a and 2b) generates two stable fixed points, i.e., (0, 1) and (1, 0), which indicate *soft* and *obey*, and *hard* and *disobey* strategies, respectively. Here, a change in the values of the feasible parameter set does not change the stability properties of the two points, but has only the effect of changing their basin of attraction.

In the first case (scenarios 1a-1d), the number of stable fixed points may vary between two, one, and zero. Two stable fixed points arise when the dominant strategy for citizens (net of the peer effect) under both hard and soft lockdowns is *obey*, but the peer effect is large. This leads to dynamics represented by a diverging spiral from the internal equilibrium that ends in one of the two stable points, exactly which one depends on the initial conditions.

One fixed point arises when i) the dominant strategy for citizens in both regimes is *disobey*; this is irrespectively of the value of the peer effect (case 1c), or ii) when citizens’ dominant strategy under soft lockdown is *disobey*, while the dominant strategy under hard lockdown (net of the peer effect) is *obey*, but the peer effect is large (case 1a). In these subcases, the diverging spiral ends in the unique stable fixed point (1, 0), that is, *hard* and *disobey*.

In some circumstances, the system may also have zero fixed points. From a mathematical point of view, when citizens are indifferent between obeying and disobeying at the two points (1, 0) and (0, 1), the two boundary equilibria $E_{b1}$ and $E_{b2}$ collapse with the two corner equilibria (0, 1) and (1, 0). Moreover, the latter inherits the stability properties of the former (i.e., we have an edge-to-corner bifurcation). This implies that the system perpetually rotates around the internal equilibrium (Fig. 5).

As stated in Introduction, our initial research question was to explain the cross-country differences observed in the governments’ management of the pandemic, given similar infection levels. However, our model may also provide a hint on what could happen within a single country over time. For example, the “diverging spiral” that describes the system dynamics as shown in case 1 above may justify the containment policy implemented by the Italian Government from September 2020 through April 2021. Indeed, the Government reclassified regions and municipalities according to the intensity of the spread of the infection and assigned them different colors accordingly. This classification was periodically revised (weekly or every two weeks, and even more often under some circumstances, e.g., the Christmas period, when harder policies were implemented to counter-balance the higher incentives to disobey). Each color represented a different degree of strictness in the lockdown imposed (red = hard lockdown; orange = medium lockdown; and yellow = soft lockdown). Over the two-year Pandemic period, all Italian regions and municipalities alternated short periods of hard policies with other periods of soft policies, and their dynamics resembled those shown in Figs. 2, 3, or even 5. Fortunately, with the mass public vaccination policy that began in late February 2021, the dynamic situations changed dramatically in favor of a more stable dynamic characterized by obeying citizens and relatively soft policies.

Our model predicts that incentives determine whether we can find two, one or zero stable fixed points and, in the case of two steady states, their basin of attraction. When citizens’ incentives are such that the dominant strategy is always *disobey* (or, in alternative, when the peer effect is large enough to offset the excess utility of obey compared to disobey under a hard policy), the system allows a unique stable state *hard* and *disobey*. If we assume that the two stable fixed points exist, i.e., *hard* and *disobey*, and *soft* and *obey*, and that the first equilibrium is not socially desirable, the question that a politician may ask himself is how to coax the system to end in an equilibrium that is more desirable from a social point of view, i.e., in which all citizens obey and politicians choose a soft lockdown policy. On the other hand—from our point of view—the question is “How is this equilibrium more likely to emerge”? In other words, how is it possible to increase its basin of attraction or implement policies that modify the parameter values of citizens’ payoff functions, to increase the chance that a desirable outcome emerges? A general answer is to increase the payoff of obeying citizens while reducing the payoff of the non-obeying ones. Several ways could be pursued to reach this goal. One would be to implement policies aimed at increasing protection against the virus for those who obey. Alternatively it could be possible to compensate the economic costs of obeying for those who do obey. The latter would be to avoid cost considerations from prevailing over the individual decision to not obey. Indeed, from an empirical point of view, it is true that higher levels of compliance with lockdown rules were attained in countries where the population is richer or the government mitigated the economic costs of isolation, such as in Austria and Iceland (Wright et al. 2020). Moreover, a government should approve and enact policies aimed at assuring the transiency of the losses that lockdown policies may generate. The fear of a permanent loss of one’s job (and consequently a permanent loss of future income) may induce citizens to disobey. Therefore, policies aimed at sustaining firms, who do not fire their employees, may help to reduce $C_i$. However, ambiguous effects on the chance of reaching the desirable outcome (0, 1) may be due to how governments modulate fines and the length of the lockdown policy. Indeed, if increasing the expected fine is a way of decreasing the payoff of the non-obeying citizens—either by increasing the fine value *F* or the probability of catching non-obeying citizens (i.e., by increasing controls of compliance with the rules), it is also true that an increase in the expected fine induces politicians to choose a hard lockdown, instead of a soft one. A similar result can be obtained for the duration of a policy. If, on one hand, the length ${\bar{L}}$ increases the psychological benefit of disobeying and increases the economic cost of obeying, on the other, it increases the probability that a politician might choose a hard lockdown policy. Therefore, these effects may compensate one another. An issue (optimal fines versus optimal length of the lockdown) that remains for future research.

## Concluding remarks

In this paper, we developed a noncooperative evolutionary game with two populations of politicians and citizens which can rationally explain the evidence that similar countries with similar levels of infection implemented very different containment policies. This model can also justify why countries with different levels of infection adopted very similar containment policies, and—moreover—why we may observe a rapid alternation of hard and soft measures within a single country and a short period of time. Our model is interesting from a dynamics point of view because it offers a rationale for a variety of short-term policies which characterized the months in which the COVID-19 pandemic was at its relative peak.

Incentives faced by the populations of citizens are important for determining the dynamics of the system. We identify six different scenarios according to citizens incentives, each one leading to a different dynamic in the existent stable steady states. If politicians maximize their payoffs by searching for popularity or by maximizing revenues through fines, the economic incentives of citizens and the peer effect play a crucial role in determining the number of fixed points, their stability properties, and the dynamics converging to them. In particular, when citizens’ excess utility of disobey compared to obey is lower following politicians’ choice of a hard confinement policy over a soft one, an oscillatory dynamic arises that is characterized by rapid changes in the strictness of the confinement policy and on the level of citizens’ compliance. Diversely, the system converges uniformly toward the attractive fixed point, depending on the initial conditions.

Economies providing different individual incentives may therefore generate different dynamics which—in the short run—may rationally explain the evidence cited above which also motivated this research paper.

In the “long term,” our analysis shows that, depending on the parameter set, the two strategies of citizens and politicians converge toward either hard policy and disobey or soft policy and obey (in the case in which two steady states coexist), or to hard policy and disobey (in case of the existence of only one steady state). Under certain circumstances, the system may be globally unstable and continue rotating around the interior equilibrium. Convergence toward the stable fixed point or the perpetual oscillation around the interior equilibrium can only be avoided by an “exogenous shock” such as a massive vaccination campaign, for example.

We are aware that when setting up the model, we made some assumptions that might be debatable for some readers. One of those is certainly the hypothesis that a soft lockdown is a more popular policy than a hard lockdown, which in our model implies $\omega _s>\omega _h$. Indeed, in some countries the contrary could be true. During his presidency, Donald Trump continued to reject the possibility of implementing a lockdown policy in the USA, claiming that the cure for the coronavirus—meaning the economic costs of the lockdown (which he claimed would be around 15 billion dollars per day)—would have been worse for the country than the cost of lives lost. As is noted, several months later Trump lost his reelection bid; thus, a lockdown-related decision undoubtedly played a significant role in the 2020 presidential elections in the USA. Similarly, in the UK, for example, if—at the beginning of the pandemic the P.M. Boris Johnson ruled out any hard policy on lockdown—following the collapse of his popularity due to the increasing number of deaths, he rapidly changed his mind and put Britain under a hard curfew. He announced that this would last while the UK waited for the availability of vaccines. A relaxation of this hypothesis may possibly generate new equilibria or different stability properties of the equilibria generated by our model, which may explain even better the differences existing between countries concerning policies on the rate of infection. We believe that this work can represent a valuable starting point for future investigation.

On a final but not less important note, we must point out that we did not study the evolution of the infection rate, which is assumed herein to be given and constant over time. This variable was set for mathematical tractability and to avoid diverting attention from the rationale governing the variegated policies observed at a global level. Studying the evolution of the infection rate under this setup could imply incorporation of the evolutionary selection mechanism of citizens’ and politicians’ choices into an SIR model; however, for the reasons outlined above, such a move would go beyond the scope of this manuscript. Nevertheless, it could undoubtedly represent a possible topic for future research.

## Appendix: Existence of the Equilibria and Stability Analysis

First of all, we will compute the equilibrium points that exist in the system 23. Setting out ${\dot{x}}_{h}=0$ and ${\dot{y}}_{O}=0$, it is easy to see that the two equations are simultaneously solved for the four corner solutions (0,0), (0,1), (1,0) and (1,1).

The other non-corner equilibria can be easily computed with few manipulations of the equations in the system. Let us rewrite the system 23, with ${\dot{x}}_{h}=0$ and ${\dot{y}}_{O}=0$:

$$\begin{aligned} \left\{ \begin{array}{l} x_h(1-x_h)\left[ {\bar{L}}y_O(\omega _s-\omega _h) + F(1-y_O)(\sigma _s-\sigma _h)\right] =0 \\ \\ y_O (1-y_O)\left[ x_h(B-C_h{\bar{L}})+ (1-x_h)(\mu B-C_s{\bar{L}})-{\bar{L}}(\beta -x_h(1-\beta ))+\theta D- \right. \\ \quad \quad \left. \gamma (1-y_O)+F(\sigma _hx_h+\sigma _s(1-x_h)) \right] ] =0 \end{array} \right. \end{aligned}$$

If we allow $x_h$ to be equal to zero, it is clear that the first equation of the system is satisfied for any feasible value of $y_O$, but not the second. If we substitute $x_h=0$ in the second equation of the system, we get that:

$$\begin{aligned} y_O (1-y_O) [(\mu B-C_s{\bar{L}}-{\bar{L}}\beta +\theta D +F \sigma _s -\gamma (1-y_O) ] =0 \end{aligned}$$

with solution equal to

$$\begin{aligned} y_O= \frac{{\bar{L}}( \beta + C_S)- \mu B -D \theta -F \sigma _s+\gamma }{\gamma }) \end{aligned}$$

So, another solution (a boundary solution), that we have defined as $E_{b_1}$ is equal to

$$\begin{aligned} E_{b_1}=\Bigg (0, \bigg ( \frac{{\bar{L}}( \beta + C_S)- \mu B -D \theta -F \sigma _s+\gamma }{\gamma }\bigg ) \Bigg ) \end{aligned}$$

Similarly, if we assume that $x_h=1$, it is clear that, once again, the first equation of the system is satisfied for any feasible value of $y_O$. The second equation instead becomes

$$\begin{aligned} y_O (1-y_O) [(B-C_h{\bar{L}} - {\bar{L}}+\theta D +F \sigma _h -\gamma (1-y_O) ] =0 \end{aligned}$$

with solution equal to

$$\begin{aligned} y_O=\frac{{\bar{L}}(1+C_h) -B - \theta D -F \sigma _h +\gamma }{\gamma } \end{aligned}$$

Therefore, the second boundary solution to the system, which we have defined $E_{b2}$, is equal to

$$\begin{aligned} E_{b2}=\Bigg (1, \bigg ( \frac{{\bar{L}}(1+C_h) -B - \theta D -F \sigma _h +\gamma }{\gamma } \bigg ) \Bigg ) \end{aligned}$$

The last (internal) equilibrium can be easily computed by dividing both right- and left-hand sides of the first and the second equations, respectively, for $x_h(1-x_h)$ and $y_O(1-y_O)$. After some rearrangements, we can express the first equation as:

$$\begin{aligned} y_O=\frac{F(\sigma _s - \sigma _h)}{F(\sigma _s - \sigma _h) - L(\omega _s - \omega _h)} \end{aligned}$$

which, substituted into the second, after some manipulations yields the already known result for $x_h$:

$$\begin{aligned} x_h= \frac{-{\bar{L}}(\beta + C_s) +\theta D +\mu B - (1-y_O)\gamma +\sigma _s F}{{\bar{L}}(1-\beta ) + B(\mu -1)+{\bar{L}}(C_h-C_s)-F(\sigma _h-\sigma _s)} \end{aligned}$$

Therefore, another equilibria for the system are

$$\begin{aligned} \Bigg ( \bigg ( \frac{-{\bar{L}}(\beta + C_s) +\theta D +\mu B - (1-y_O)\gamma +\sigma _s F}{{\bar{L}}(1-\beta ) + B(\mu -1)+{\bar{L}}(C_h-C_s)-F(\sigma _h-\sigma _s)}\bigg ), \bigg ( \frac{F(\sigma _s - \sigma _h)}{F(\sigma _s - \sigma _h) - L(\omega _s - \omega _h)}\bigg ) \Bigg ) \end{aligned}$$

Summarizing, we can claim that the system admits the existence of at most seven fixed points. Now, we assess whether the seven equilibria are *asymptotically stable (unstable) points* by analyzing the *Jacobian Matrix* $J\left( {\dot{x}}_{h},{\dot{y}}_{O}\right)$ computed at the equilibrium points. The local stability of the each equilibrium point is determined through the usual linearization procedure, i.e., according to the study of the eigenvalues of the Jacobian matrix. The idea is to use the eigenvalues of the Jacobian matrix evaluated at a critical point to understand the behavior of the system near that critical point (Bischi et al., 2015). For the RD system (23), its Jacobian matrix is given by:

$$\begin{aligned} J\left( {\dot{x}}_{h},{\dot{y}}_{O}\right) = \begin{pmatrix} J_{11} &{} J_{12} \\ J_{21} &{} J_{22} \end{pmatrix} \end{aligned}$$

where

$$\begin{aligned} J_{11}= & {} (2 x^*-1) \left( (y^*-1) F (\sigma _h - \sigma _s)+ y^* {\bar{L}} \left( \omega _s-\omega _h\right) \right) \\ J_{12}= & {} x^* (x^*-1) \left( F (\sigma _h- \sigma _s)+{\bar{L}} \left( \omega _s-\omega _h\right) \right) \\ J_{21}= & {} y^* (y^*-1) \left( {\bar{L}}(1- \beta ) -B (1-\mu )+{\bar{L}}( C_H- C_S)-F \left( \sigma _h-\sigma _s\right) \right) \\ J_{22}= & {} (2y^* -1)\bigg (x^*({\bar{L}}-B +{\bar{L}}C_H - F \sigma _h) + (1-x^*)({\bar{L}}\beta - B\mu +{\bar{L}}C_S - F \sigma _s) \\{} & {} - \theta D \bigg ) - (y^*-1)(3y^*-1)\gamma \end{aligned}$$

The stability analysis at the corner equilibrium, as well as at the boundary equilibria, is straightforward, as the Jacobian matrix is diagonal at any corner equilibrium and triangular at any boundary equilibrium, and hence, in both cases the eigenvalues are given by the diagonal entries.

Let us analyze the equilibria one by one, rearranging according the notation introduced in Eqs. 14–17. That is:

- At the equilibrium point (1, 1),
  $$\begin{aligned} J( 1,1) = \begin{pmatrix} {DL} &{}\,\,\, 0 \\ 0 &{}\,\,\, {W} \end{pmatrix} \end{aligned}$$
  In this situation, we can easily prove that the eigenvalues of *J*(1, 1) are equal to $\lambda _1 = DL$ and $\lambda _2=W$. By definition, *DL* is positive, so is $\lambda _1$. Depending on the choice of the parameters, $\lambda _2$ could be either positive or negative. This means that at least one (and possibly two) eigenvalues are positive, which make this fixed point asymptotically unstable.
- At the equilibrium point (0, 0), we have:
  $$\begin{aligned} J( 0,0) = \begin{pmatrix} {DF} &{}\,\,\, 0 \\ 0 &{}\,\,\, {-K-\gamma } \end{pmatrix} \end{aligned}$$
  In this case, it is trivial to compute the eigenvalues, since they are equal to $\lambda _1 = DF$ and $\lambda _2 = - K - \gamma$. As said before, $\lambda _1$ is always positive by definition, so, whatever the sign of *K*, this point has always at least one eigenvalue positive, which make this equilibrium asymptotically unstable.
- At the equilibrium point (0, 1), we have:
  $$\begin{aligned} J( 0,1) = \begin{pmatrix} {-DL} &{}\,\,\, 0 \\ 0 &{}\,\,\, {K} \end{pmatrix} \end{aligned}$$
  The eigenvalues here are trivial to compute. The first eigenvalues $\lambda _1$ are equal to $\lambda _1 = -DL$, which is negative by definition, while $\lambda _2=K$, which might be positive or negative. If $K<0$, that is to say, if the excess utility of non-obey with respect to obey under soft lockdown, net of the peer effect, is negative, then the equilibrium is asymptotically stable.
- At the equilibrium point (1, 0), we have:
  $$\begin{aligned} J( 1,0) = \begin{pmatrix} {-DF} &{}\,\,\, 0 \\ 0 &{}\,\,\, {-W-\gamma } \end{pmatrix} \end{aligned}$$
  Once again, it is trivial to show that $\lambda _1 = -DF$ and $\lambda _2=-W-\gamma$. $\lambda _1$ is negative by definitions, since the conditions on the parameters were such that $\sigma _h>\sigma _s$. Moreover, depending on the sign and size of *W*, if the excess utility of non-obey with respect to obey under hard lockdown (net of the peer effect) is greater than the peer effect, then also $\lambda _2$ is negative, and this condition guarantees that this equilibrium is asymptotically stable.
- At the border equilibrium $E_{b1}=(0, \frac{{\bar{L}}( \beta + C_S)- \mu B -D \theta -F \sigma _s+\gamma }{\gamma }) = (0, \frac{K+\gamma }{\gamma })$, the Jacobian is:
  $$\begin{aligned} J( E_{b1} ) = \begin{pmatrix} J_{11} &{}\,\,\, 0 \\ J_{21} &{}\,\,\, J_{22} \end{pmatrix} \end{aligned}$$
  Exploiting Eqs. 14–17 and manipulating a bit, we obtain that:
  $$\begin{aligned} J_{11}= & {} - \frac{K \cdot DF+(\gamma + K)\cdot DL}{\gamma } \\ J_{21}= & {} \frac{K(\gamma + K)\cdot (W-K)}{\gamma ^2} \\ J_{22}= & {} - \frac{K(\gamma +K)}{\gamma } \end{aligned}$$
  Since the matrix is triangular, it is easy to prove that $\lambda _1 = J_{11}$ and $\lambda _2=J_{22}$. The analysis of the sign of $J_{11}$ and $J_{22}$ is therefore crucial to determine the sign of the eigenvalues and therefore the stability of the border equilibrium $E_{b1}$. It is easy to see that $J_{11}$ is negative if two conditions hold, alternatively: a) $K>0$ or b) $K<0$ and $\gamma > \frac{K(DL+DF)}{DL}$, and it is positive if $K<0$ and $0<\gamma < \frac{K(DL+DF)}{DL}$. At this border equilibrium $E_{b1}$, it is easy to see that $J_{22}$ is negative when $K>0$ or when $K<0$ and $0<\gamma < -K$ (i.e., the peer effect is “small enough”) and positive otherwise. Moreover, it can be easily noticed that at this corner equilibrium $J_{22}$ can be rewritten as:
  $$\begin{aligned} J_{22}= -K \cdot y_O^* \end{aligned}$$
  which is always negative when $K>0$ and positive otherwise. Recall that $y_O^*$ is always positive since it is included between 0 and 1. It follows that if $K>0$, the equilibrium is stable, but it lies outside the strategy space of the players and then it does not exist as a feasible outcome. If, instead, $K<0$, we can claim the presence of a saddle point only if the condition
  $$\begin{aligned} -K \cdot \frac{DL+DF}{DL} < \gamma \end{aligned}$$
  holds, while in the other case we can state that $E_{b1}$ is an unstable point.
- At the border equilibrium $E_{b2}=(1, \frac{{\bar{L}}(1+C_h) -B - \theta D -F \sigma _h +\gamma }{\gamma }) =(1, \frac{W+\gamma }{\gamma })$, the Jacobian matrix is:
  $$\begin{aligned} J( E_{b2}) = \begin{pmatrix} J_{11} &{}\,\,\, 0 \\ J_{21} &{}\,\,\, J_{22} \end{pmatrix} \end{aligned}$$
  where, making use of Eqs. 14–17, then:
  $$\begin{aligned} J_{11}= & {} \frac{W \cdot DF + DL \cdot (W+\gamma )}{\gamma } \\ J_{21}= & {} \frac{W(\gamma +W)}{\gamma ^2}\cdot (W-K) \\ J_{22}= & {} -\frac{W(\gamma +W)}{\gamma } \end{aligned}$$
  The characteristic polynomial of $J(E_{b2})$ is $\lambda ^2 - \lambda (J_{11}+J_{22})+ J_{11}J_{22}=0$ which leads to the following roots (eigenvalues): $\lambda _1=J_{22}$ and $\lambda _1=J_{11}$. The sign of $J_{11}$ and $J_{22}$ is therefore crucial to determine the stability of this border equilibrium $E_{b2}$. It is easy to see that $J_{11}$ is positive when *W* is positive, while if *W* is negative, $J_{11}$ is negative when $\gamma < -\frac{W (DL + DF)}{DL}$, that is to say, when the peer effect is “small enough.” Moreover, if *W* is positive, it implies that the border equilibrium lies outside the strategy space of the players and therefore we must impose *W* negative as a constraint. Similarly to what has been done with the previous border equilibrium, with little manipulations we can rewrite $J_{22}$ as:
  $$\begin{aligned} J_{22} = - W \cdot y_o^* \end{aligned}$$
  whose sign depends exclusively on *W*. Therefore, $J_{22}$ is negative when *W* is positive and vice versa. So, in order to be stable, the border equilibrium $E_{b2}$ must have two negative eigenvalues associated with his Jacobian. However, the values of *W* that guarantee the simultaneous negative sign of $J_{11}$ and $J_{22}$ cannot coexist, because $J_{11}$ is negative for negative values of *W* and sufficiently small peer effect, while $J_{22}$ is negative when *W* is positive. It follows that the border equilibrium $E_{b2}$ might be a saddle point, when the peer effect is small; otherwise, it is globally unstable.
- The interior equilibrium $(x^*_h, y^*_O)$ is defined as
  $$\begin{aligned} (x^*_h, y^*_O) =\bigg ( \frac{K + \gamma \big (1- \frac{DF}{DF+DL}\big )}{K-W}, \frac{DF}{DF+DL}\Bigg ) \end{aligned}$$
  , and at this equilibrium point, the corresponding Jacobian matrix is defined as:
  $$\begin{aligned} J( x^*_h, y^*_O) = \begin{pmatrix} 0 &{}\,\,\, J_{12} \\ J_{21} &{}\,\,\, J_{22} \end{pmatrix} \end{aligned}$$
  where
  $$\begin{aligned} J_{12}= & {} \frac{DF + DL}{(W-K)^2}\cdot \big ( K+\gamma (1-\frac{DF}{DF+DL})\big )\cdot \big ( W+\gamma (1-\frac{DF}{DF+DL}) \big ) \\= & {} \frac{DF + DL}{(W-K)^2} \cdot \Bigg ( \gamma ^2 \bigg ( \frac{DL}{DF+DL}\bigg )^2 + \gamma \bigg ( \frac{DL(K+W)}{DF+DL}\bigg ) + KW \Bigg ) \\ J_{21}= & {} - \frac{DF \cdot (W-K) \cdot DL}{(DF + DL)^2} \\ J_{22}= & {} \frac{\gamma \cdot DF \cdot DL}{(DF + DL)^2} \end{aligned}$$
  The eigenvalues of this matrix can be computed by solving for $\lambda$ the characteristic polynomial given by the following expression:
  $$\begin{aligned} \lambda ^2 - \lambda J_{22} - J_{21}J_{12}=0 \end{aligned}$$
  which yields the solutions as:
  $$\begin{aligned} \lambda _{1,2}= \frac{J_{22} \pm \sqrt{J_{22}^2 + 4 J_{21}J_{12}}}{2} \end{aligned}$$
  Now, it easy to realize that $J_{22}$ is always positive for any feasible value of the parameters. $J_{21}$ instead is positive when $(W-k)$ is positive, and negative otherwise. In order, this equilibrium exists, *W* and *K* must be both negative or both positive. (If *W* and *K* have opposite sign, the equilibrium is not included in the feasible strategy space, and moreover, $|W|>|K|$.) The fact that $J_{22}$ was positive implies that the eigenvalues of this Jacobian matrix cannot be contemporaneously negative, conditions under which stability is guaranteed. If indeed $J_{22}^2 + 4 J_{21}J_{12}$ is positive, we have a saddle point, while if $J_{22}^2 + 4 J_{21}J_{12}$ is negative, we will have two complex eigenvalues with positive real part. Let us develop the conditions for that to happen. With some manipulations, the expression $J_{22}^2 + 4 J_{21}J_{12}$ can be rewritten as a function of $\gamma$ as follows:
  24 $$\begin{aligned} J_{22}^2 + 4 J_{21}J_{12}= & {} \gamma ^2 \cdot \big [y{^*}{^2} (1-y^{*})^2 -4(y^* (1-y^*)^3 (W-K) Z)\big ] + \nonumber \\{} & {} - \gamma \big [4 y^* (1-y^*)^2 (K+W)(W-K)Z\big ] + \nonumber \\{} & {} - 4y^*(1-y^*)(W-K)ZKW \end{aligned}$$
  where
  $$\begin{aligned} Z=\frac{DF+DL}{(W-K)^2}>0 \end{aligned}$$
  It is easy to see that the coefficient attached to $\gamma ^2$ might be positive or negative depending on the difference between the excess of utility of non-obey with respect to obey in hard and in soft lockdown. If this difference, represented by $W-K$, is negative, it is easy to show that the coefficient attached to $\gamma ^2$ is positive, and this implies that if the peer effect is small enough, there exist two real eigenvalues for the Jacobian associated with this equilibrium, one positive and one negative, making the equilibrium an unstable saddle node. Solving Eq. 24 for $\gamma$, indeed the solution of the inequality $J_{22}^2 + 4 J_{21}J_{12}>0$, which guarantees real eigenvalues, is internal to the roots. In indeed the excess of utility of disobey in hard lockdown is greater than in soft lockdown; that is, if $W-K>0$, the sign of the coefficient attached to $\gamma ^2$ is ambiguous. If it is still positive, the same conditions stated before hold (we have a saddle point); otherwise, we will have two complex eigenvalues with positive real part, which leads the system rotate around this equilibrium, but converging to either one of the two stable equilibria (0,1) or (1,0). Solving Eq. 24 for $\gamma$ and rearranging, taking into consideration the expression for *Z* we get the following roots:
  $$\begin{aligned} \gamma _1= & {} \frac{2 \cdot \bigg (-(K+W)(1-y)^2 \cdot \frac{DF+DL}{W-K} +\sqrt{(1-y)^3 \cdot \frac{DF+DL}{(W-K)}\cdot [WKy + (1-y)(W-K)(DF+DL)]} \bigg )}{(1-y)^2 \big (\frac{4\cdot DL}{W-K} - \frac{DF}{DF+DL} \big )} \\ \gamma _2= & {} \frac{2 \cdot \bigg (-(K+W)(1-y)^2 \cdot \frac{DF+DL}{W-K} -\sqrt{(1-y)^3 \cdot \frac{DF+DL}{(W-K)}\cdot [WKy + (1-y)(W-K)(DF+DL)]} \bigg )}{(1-y)^2 \big (\frac{4\cdot DL}{W-K} - \frac{DF}{DF+DL} \big )} \end{aligned}$$
  It is easy to see that if $W-K<0$, both roots are real, and the solutions of the inequality lie at the interior of the interval between $\gamma _1$ and $\gamma _2$. Moreover, these conditions guarantee that the eigenvalues associated with the Jacobian are real. In particular, in order to have real eigenvalues, the peer effect has to be smaller “enough,” that is to say, it has to be included between 0 and the greater between $\gamma _1$ and $\gamma _2$, depending on the sign and dimension of *K*. If these conditions are not met, that is to say, if the peer effect is greater than the threshold, then the eigenvalues are complex with positive real part, and this implies that the system will diverge from the interior equilibrium following an enlarging spiral toward one of the two stable equilibria (0,1) or (1,0).

## Footnotes

1 The Government Response Stringency Index is a composite measure based on nine response indicators including school closures, workplace closures, and travel bans, rescaled to a value from 0 to 100 (100 = strictest response). The biweekly growth rate on any given date measures the percentage change in the number of new confirmed cases over the last 14 days relative to the number in the previous 14 days. https://ourworldindata.org/grapher/government-response-stringency-index-vs-biweekly-change-in-confirmed-covid-19-cases.

2 The Guardian: https://www.theguardian.com/commentisfree/2020/sep/23/freedom-loving-brits-prime-minister-state-conservative, https://www.theguardian.com/world/2020/sep/22/johnson-refuses-to-rule-out-second-covid-19-lockdown-amid-new-restrictions.

3 Dhami et al. (2020) point out that “subjective experiences of lockdown were comparable to those of first-time prisoners” where “males in lockdown were equally often accused of disobeying the rules of lockdown as were first-time prisoners in being charged with misconduct in prison,” and thus indicating the extent of psychological costs associated with a policy of strict confinement.

4 The literature and research articles on COVID-19 have evolved a lot over the last two years [among others see Bosi et al. (2021), La Torre et al. (2021), Powell (2022); and see also the survey paper by Brodeur et al. (2021)].

5 As we have already anticipated, we do not study the dynamics of the infection levels and we assume that these levels are given and observed by politicians. Our main goal is indeed to justify the coexistence of very different containment policies for a given level of the infection rate, and not to investigate the determinants of the evolution of the infection.

6 For example, a hard lockdown can be thought of as a lockdown that involves all productive activities but those essential for survival, while a soft lockdown can be thought of as a lockdown that leaves all productive activities to do business as usual, but with some limitations. For instance, a hard lockdown may demand that any restaurant be shut down even for short time, while a soft lockdown may demand that only a percentage of customers have access to the restaurant.

7 Some economists, too, argued that from an economic point of view, even an extreme pandemic was better than the lockdown in Australia. Economist Gigi Foster estimated the impact of a lockdown on multiple spheres concerning people’s well-being and claimed—not without harsh criticisms—that Australian people, in her opinion, would have been better off without a lockdown.

8 These assumptions are introduced for mathematical tractability. There are indeed several indicators suggesting that both *B* and $C_i$ might not be the same across individuals. For instance, it was found that Black Brazilians were twice as likely as white Brazilians to have had COVID-19 symptoms. At the same time, they were likelier to suffer economic consequences of pandemic in the form of job losses or pay cuts (The New York Times, https://www.nytimes.com/article/brazil-coronavirus-cases.html). Also, the King’s College of London’s survey reveals that some of the reasons for non-compliance are “going out to work” and caring for others’ need (the King’s College London survey in Bloomberg Opinion, https://www.bloomberg.com/opinion/articles/2020-09-30/covid-19-disobedience-goes-deeper-than-we-think, MoveHub https://www.movehub.com/blog/best-and-worst-covid-responses/). However, citizens seem to obey when economic losses due to quarantines are compensated like in Austria and Iceland (the King’s College London survey in Bloomberg Opinion, https://www.bloomberg.com/opinion/articles/2020-09-30/covid-19-disobedience-goes-deeper-than-we-think).

9 It is indeed confirmed by the medical literature that a reduction of social contacts is associated to an increase of mental health impairments (Christoph et al. 2020; Murphy et al. 2020). Moreover, Tunçgenç et al. (2021:13) found evidence of peer effect in the compliance to the rules by individuals.

10 $D=0$ means that those who become infected are asymptomatic, therefore their loss of health is null.

11 Peter D. Taylor and Leo Jonker in 1978 published their seminal paper, entitled “Evolutionarily stable strategies and game dynamics,” in which the mainly adopted dynamic foundation of evolutionary dynamics is the one known as “replicator dynamics,” explicitly expressed through differential or difference equations, thus recognizing the close links between a game-theoretic approach and the theory of dynamical systems. See https://doi.org/10.1016/0025-5564(78)90077-9.

12 These two fixed points exist in the action space of the players only for a subset of the possible combinations of the feasible parameters’ set.

13 Local stability of an equilibrium point means that if you put the system somewhere nearby the point, then it will move itself to such an equilibrium point in some time. An equilibrium that is stable “in most cases” is an equilibrium that is stable in the majority of the scenarios.

14 A qualitatively similar dynamics would be obtained with $\gamma$ smaller than *W*, which would have guaranteed a dominant strategy under hard policy equal to obey, irrespective of the value of the peer effect.
